# The Use of Secret Preparations

**Published:** 1901-06-15

**Authors:** 


### THE USE OF SECRET PREPARATIONS.

Complaint is made from time to time of our use of secret preparations, amalgam, etc., etc. I do not think we fall below the true professional standard in this more than do our medical friends. It is not an uncommon thing for a medical man to patent some combination of medicine and be excommunicated, and then for the rank and file of regular physicians to go on prescribing the patented medicine.

Such medicines were prescribed several times in my family by the family physician, and when I pointed out the seeming inconsistency, his reply was that he knew what the combination contained, and it was a good one. I think the same holds true for the most part in our profession. I think we are not to any great extent in the habit of using preparations, the ingredients of which we do not know.

A bad medicine will have a bad effect, and that will limit or terminate its use. No one, unless he be a fool or a knave, can afford to use a bad medicine very long. In the matter of amalgams it seems to me there is no great danger to the public or to our profession, if the ingredients be not known. I see no reason why a dentist should know the exact proportions of the metals used in an amalgam—and that is all there is that is kept secret. Everybody knows what amalgams are made of, and the profession could not be long cheated by the use of deleterious metals. The perfect working of the machinery of modern life depends upon faith. Men must trust each other or the business of the world must stop. Some one must be trusted to make the amalgams we use. To make them ourselves would be like going back to barbarism, the keynote of which was distrust. A dentist who can make his own amalgam and clean his hands after it can not have a very large clientele. In my judgment there is no need of this anxiety, for the dentist who uses the amalgam is the master of the situation. He is the best and the final judge of its quality. If it is poor he will not use it; if it is the best, he will have no other. I think the profession ought to be glad when a practical dentist of vast experience and spotless reputation is willing to turn aside from his professional work and make a long series of expensive experiments for the purpose of making a better amalgam. Such a man should be a competent judge of what constitutes a good amalgam, and I do not think he is called upon to give to the public his formula. If he does, anybody can make his amalgam, and make it poorly. His rivals might be glad to eliminate him in that way. If he keeps his secret, no one has any right to sell any amalgam as his, and in that way, and in that way only, can he protect himself and his profession. To make and sustain an amalgam of such high order is to stimulate the manufacturers all along the line, and the result is apparent at once throughout the profession, for I think it is safe to say that never before have we had such perfect amalgams as during the past few years. I think it is more creditable to make a good amalgam than to make poor use of it. I think there is more need of twitting the rank and file of the profession for careless use of poor amalgam than of criticising those who are trying to make a good one. A man who has spent a long life in conspicuous professional work, and been honored in every possible way, who will turn aside for such a purpose deserves our warmest appreciation and our firm support. It may be said he expects to make it profitable. That does not lessen its value to the profession, nor detract from the credit justly due him. It is a criticism that should be made only by those who work for nothing in this world. Republics are said to be ungrateful. I often think our profession is ready to be guilty of the same selfishness. Sometimes we rouse ourselves and throw flowers on the graves of those who have been helpful to us, but we neglect to brighten their way with them while they are living and would be cheered by them.

Of course, those who hold the views I have expressed will be credited by some with having too much enthusiasm. We must expect to hear that we are not a profession, and to be told that we are on the downward track. We will be scolded by those who like to scold, and misunderstood by those who have not the wit or willingness to read between the lines. We shall have to submit to the humiliation of the sight of the ubiquitous dental showcase and the colored gentlemen in brass buttons who stand like black spiders at the mouths of the webs of the dental parlors! We shall have to be patient with long hair and bad table manners, flash jewelry and sublime egotism, narrowmindedness and Phariseeism, and the countless minor weaknesses that make men so human, but—no class of men on earth have added more to human comfort and happiness, and we can rest our case on that. "By their fruits shall ye know them!"

We *are* a profession, and one of the most useful the world has ever known, and we were never so well equipped as at the opening of this new century, and that is exactly "where we are at!"—Dr. Perry, *Items of Interest*.

